# Supplementary material for: Nicotinamide Inhibits T Cell Exhaustion and Increases Differentiation of CD8 Effector T Cells
Source: Cancers (Basel). 2022 Jan 10;14(2):323. doi: 10.3390/cancers14020323 (PMC8774026; doi:10.3390/cancers14020323)
Supplement: Supplementary file 1 [file cancers-14-00323-s001.zip › cancers-1536188-supplementary.pdf]

cancers

MDPI

Nicotinamide Inhibits T Cell Exhaustion and Increases Differentiation of CD8 Effector T Cells

Sara Alavi, Abdullah Al Emran, Hsin-Yi Tseng, Jessamy C. Tiffen, Helen Marie McGuire and Peter Hersey

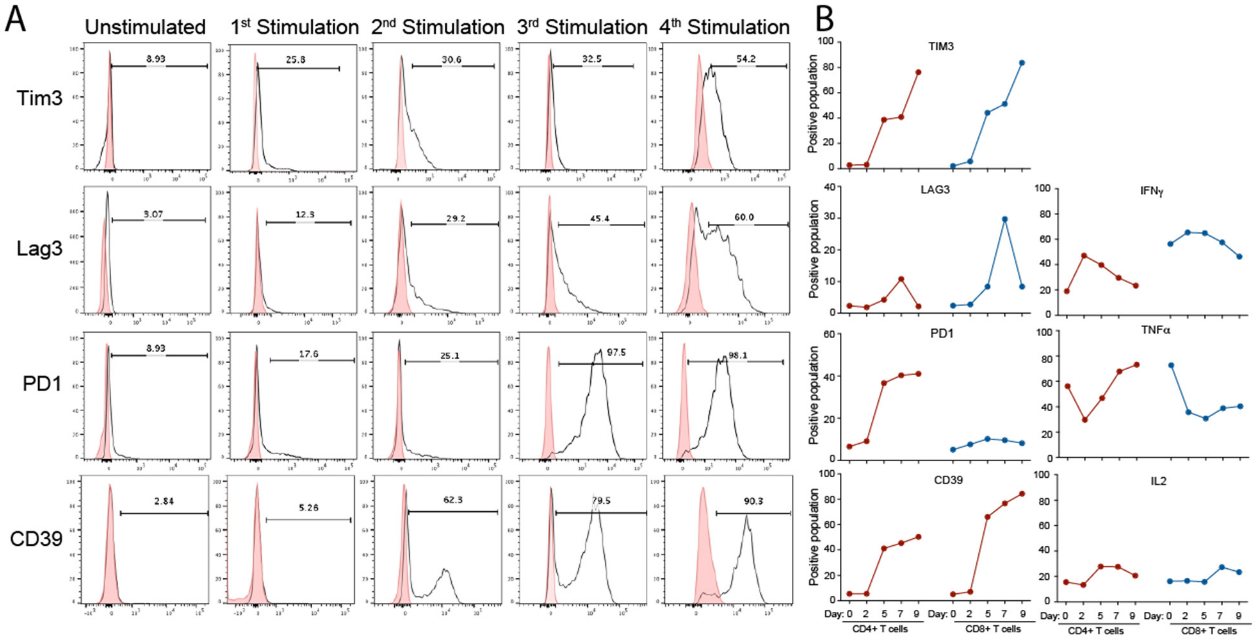

Figure S1. Robust in vitro culture system to explore T cell exhaustion phenotype. A) Persistent activation of T cells with immobilized anti-CD3 or anti-CD28 antibody shows increasing expression of exhaustion markers, PD1, LAG3, Tim3 and CD39 on CD8+ T cells. B) Time series data shows CD4+ T cells exhibited same pattern of exhaustion markers, with intracellular cytokines show high production and then decline after repeated stimulation for both CD4+ and CD8+ T cells.

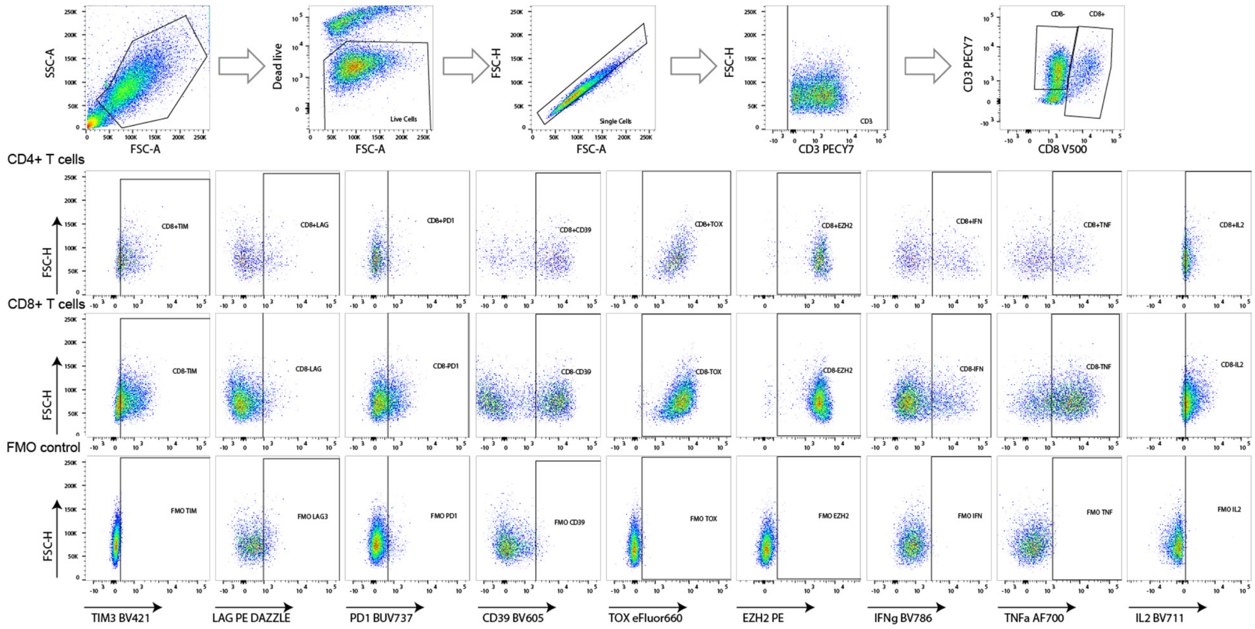

Figure S2. Gating strategy for flow cytometry assay. Bottom row refers to control samples which omitted the marker under investigation (FMO control) to guide appropriate placement of marker positive gates in both CD4+ and CD8+ T cells. Also of note, when CD3 downregulation on CD4+ T cells, inherently linked to stimulation under PMA and Ionomycin conditions deemed sequential gating on CD3+ only cells first as indeterminate (as in 4th plot in top row), all cells were included, and distinction of CD4 and CD8 T cell subsets were further clarified using CD8 co-expression, excluding CD3- CD8- cells. CD8-CD3+ cells were verified for CD4+ marker expression (data not shown).

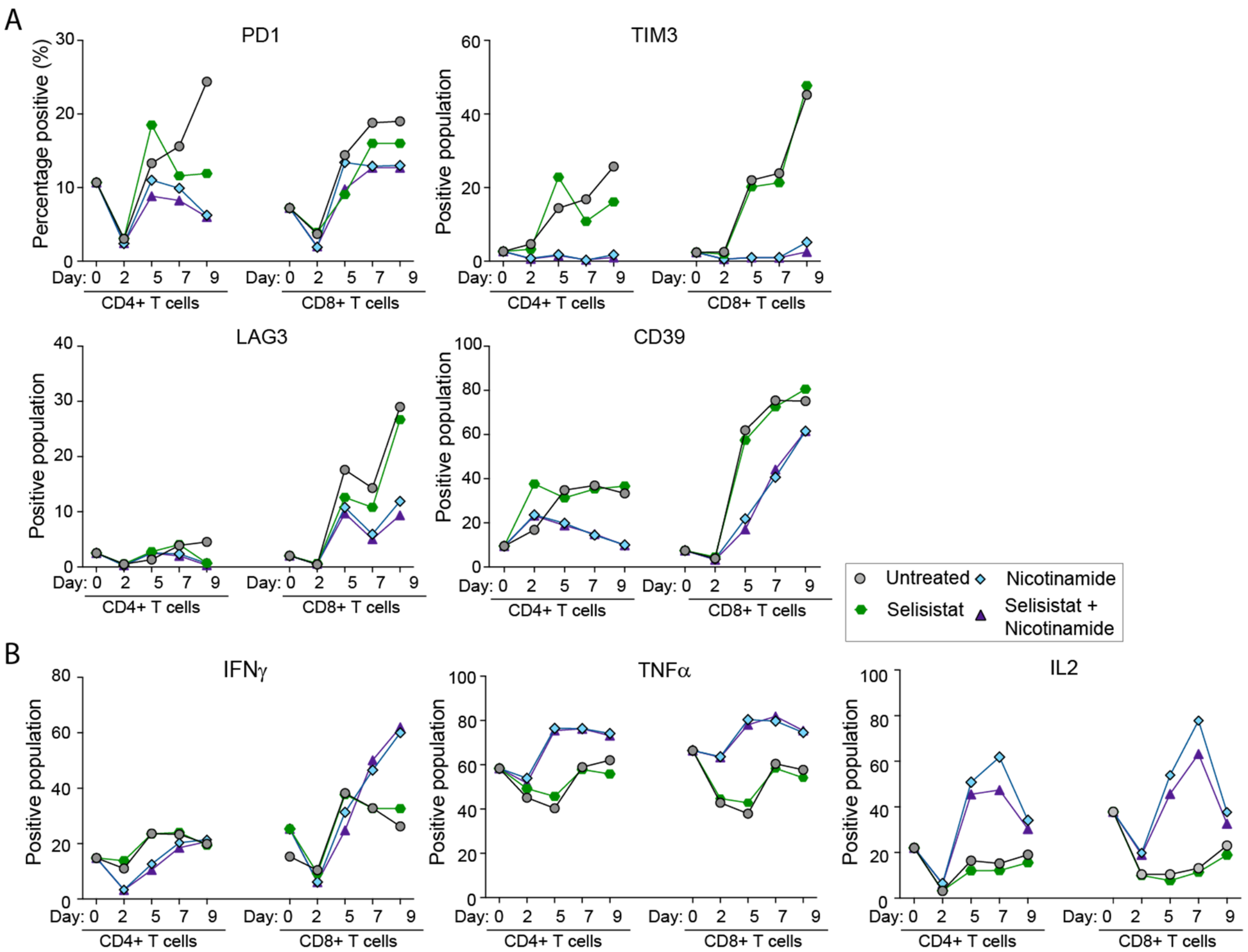

Figure S3. SIRP1 study – Sirtuins. Time course shown for representative experiment treated with Selisistat, Nicotinamide or both inhibitors showing A) series of inhibitory receptors and B) series of cytokines.

Table S1. Mass Cytometry Panel used for immunophenotyping.

Metal Isotope / Fluorochrome	Antigen	Clone	Manufacturer
Barcoding and fluorescent			
^{104}Pd, ^{106}Pd or ^{108}Pd	CD45	30-F11	BD/Biolegend
Remaining surface stain			
^{89}Y	CD8a	RPA-T8	Biolegend
^{113}In	CD56	NCAM16.2	BD
^{115}In	CD159a (NKG2A)	REA110	Biolegend
^{139}La	CD244 (2B4)	REA112	BD
^{142}Nd	CD19	HIB19	Biolegend
^{143}Nd	CD45RA	HI100	BD
^{144}Nd	CD69	FN50	Biolegend
^{145}Nd	CD4	RPA-T4	Biolegend
^{147}Sm	CD45RO	UCHL1	Sigma
^{148}Nd	CD28	CD28.2	Nobus Bio
^{149}Sm	CD366 (Tim3)	7D3	BD
^{150}Nd	KLRG1	SA231A	Biolegend
^{151}Eu	CD39	A1	Biolegend
^{154}Gd	CD3	UCHT1	BD
^{156}Gd	CD279 (PD-1)	EH12.2H7	Biolegend
^{158}Gd	CD194 (CCR4)	L291H4	Biolegend
^{159}Tb	CD223 (Lag3)	17B4	Genetex
^{163}Dy	CD183 (CXCR3)	REA232	BD
^{166}Er	TIGIT	MBSA43	eBioscience
^{167}Er	CD197 (CCR7)	G043H7	Abcam
^{168}Er	CD95	DX2	BD
^{169}Tm	CD25	M-A251	Biolegend
^{172}Yb	CD57	NK-1	Biolegend
^{173}Yb	IL-2	MQ1-17H12	BD
^{174}Yb	HLA-DR	L243	Biolegend
^{176}Lu	CD127	A019D5	Biolegend
^{209}Bi	CD27	M-T271	BD
Intracellular stain			
^{141}Pr	IFNg	B27	BD
^{146}Nd	EOMES	WD1928	BD
^{152}Sm	TNFa	Mab11	Biolegend
^{153}Eu	TOX	TXRX10	Biolegend
^{155}Gd	CD196 (CCR6)	11A9	eBioscience
^{160}Er	T-bet	4B10	
^{164}Er	GATA3	L50-823	BD
^{165}Ho	IL-10	JES3-12G8	BD
^{170}Er	TCF1/TCF7	C63D9	eBioscience
^{171}Yb	GranzymeB	REA226	Miltenyi
^{175}Lu	Perforin	dG9	Biolegend
$^{191/193}Ir$	DNA Intercalator	-	Fluidigm
